# ^18^F-alfatide PET/CT may predict short-term outcome of concurrent chemoradiotherapy in patients with advanced non-small cell lung cancer

**DOI:** 10.1007/s00259-016-3505-3

**Published:** 2016-09-08

**Authors:** Xiaohui Luan, Yong Huang, Song Gao, Xiaorong Sun, Suzhen Wang, Li Ma, Xuepeng Teng, Hong Lu, Jinming Yu, Shuanghu Yuan

**Affiliations:** 1 Department of Radiation Oncology, Shandong Cancer Hospital affiliated to Shandong University, No 440 Jiyan Road, Jinan, Shandong 250117 China; 2 School of Medicine and Life Sciences, University of Jinan-Shandong Academy of Medical Sciences, Jinan, China; 3 Department of Radiology, Shandong Cancer Hospital affiliated to Shandong University, Jinan, Shandong China; 4 Department of Oncology, Jining Infectious Diseases Hospital, Jining, Shandong China

**Keywords:** Non-small cell lung cancer, Concurrent Chemoradiotherapy, ^18^F-alfatide, PET/CT, Integrin αvβ3

## Abstract

**Purpose:**

The study aims to investigate the role of ^18^F-alfatide positron emission tomography/computed tomography (PET/CT) in predicting the short-term outcome of concurrent chemoradiotherapy (CCRT) in patients with advanced non-small cell lung cancer (NSCLC).

**Methods:**

Eighteen patients with advanced NSCLC had undergone ^18^F-alfatide PET/CT scans before CCRT and PET/CT parameters including maximum and mean standard uptake values (SUV_max_/SUV_mean_), peak standard uptake values (SUV_peak_) and tumor volume (TV_PET_ and TV_CT_) were obtained. The SUV_max_ of tumor and normal tissues (lung, blood pool and muscle) were measured, and their ratios were denoted as T/NT (T/NT_lung_, T/NT_blood_ and T/NT_muscle_). Statistical methods included the Two-example *t* test, Wilcoxon rank-sum test, Receiver-operating characteristic (ROC) curve analysis and logistic regression analyses.

**Results:**

We found that SUV_max_, SUV_peak_, T/NT_lung_, T/NT_blood_ and T/NT_muscle_ were higher in non-responders than in responders (*P* = 0.0024, *P* = 0.016, *P* < 0.001, *P* = 0.003, *P* = 0.004). According to ROC curve analysis, the thresholds of SUV_max_, SUV_peak_, T/NT_lung_, T/NT_blood_ and T/NT_muscle_ were 5.65, 4.46, 7.11, 5.41, and 11.75, respectively. The five parameters had high sensitivity, specificity and accuracy in distinguishing non-responders and responders. Multivariate logistic regression analyses showed that T/NT_lung_ was an independent predictor of the short-term outcome of CCRT in patients with advanced NSCLC (*P* = 0.032).

**Conclusions:**

^18^F-alfatide PET/CT may be useful in predicting the short-term outcome of CCRT in patients with advanced NSCLC.

## Background

Lung cancer is the leading cause of cancer death worldwide, and non-small cell lung cancer (NSCLC) accounts for 85 % [1]. One-third of patients with newly diagnosed NSCLC have been advanced and not amenable for curative resection. Concurrent chemoradiotherapy (CCRT) represents the standard of therapy protocol for patients with advanced NSCLC who have good performance status and no significant weight loss [2]. Even with the standard therapy, one-third of these patients still experience local failure [3]. Thus, it’s important to find an effective predicting tool to select patients who are likely to benefit from the treatment. This may help to personalize the treatment in NSCLC patients by avoiding ineffective CCRT and continuing the primary treatment in responding patients.

The tumor node metastasis (TNM) staging system is considered the most important prognostic tool [4], but TNM staging does not correspond to biological aggressiveness and fails to explain the wide variation of the outcomes in patients within the same stage [5]. Various techniques including all kinds of molecular imaging had been developed to predict the tumor response to therapy.

Angiogenesis, the growth of new blood vessels from preexisting vessels, is an essential step in tumor development and metastasis. It is widely accepted that the imaging of tumor angiogenesis can be used not only for the early detection of cancers but also for monitoring treatment outcomes [6]. Integrin αvβ3 has been shown to play an important role in angiogenesis and up-regulated obviously in various types of tumor cells and the activated endothelial cells of tumor angiogenesis [7–9]. Because the arginine-glycine-aspartic acid (RGD) tripeptide sequence can bind to integrin αvβ3 with high affinity and specificity [10, 11], RGD PET/CT (positron emission tomography/computed tomography) may be helpful to evaluate tumor angiogenesis. A novel one-step labeled integrin αvβ3-targeting PET probe, ^18^F-AlF-NOTA-PRGD2 (denoted as ^18^F-alfatide) has been proved to be safe [12] and can identify lung cancer clearly with desirable image contrast [13]. We had performed a pilot clinical study in which ^18^F-alfatide PET/CT parameters could predict the tumor sensitivity to CCRT in patients with glioma [14]. Therefore, we think ^18^F-alfatide PET/CT might be a potential tool for predicting the short-term outcome of CCRT in patients with advanced NSCLC.

In this clinical study, we aim to investigate whether ^18^F-alfatide PET/CT parameters could be used as a classifier for predicting the short-term outcome of CCRT in patients with advanced NSCLC.

## Materials and methods

### Patients

Eighteen patients with advanced NSCLC were enrolled in this study (Table 1). There were 14 males and four females with median age of 62 (range: 45–85). All patients had given informed consent to participate in this study, which was approved by the ethics committee of Shandong Cancer Hospital affiliated to Shandong University and met the following inclusion criteria: (1) advanced NSCLC diagnosed by histological and imaging examination such as CT or ^18^F-fluorodeoxyglucose (FDG) PET/CT (stage IIIA, IIIB or IV); (2) Karnofsky performance status (KPS) ≥70; (3) had measurable primary tumors according to Response Evaluation Criteria in Solid Tumors (RECIST). All patients were ready to undergo CCRT without undergoing surgery, chemotherapy or radiotherapy for thoracic tumors formerly, and they had the ^18^F-alfatide PET/CT scans before CCRT.

**Table 1:** Clinicopathological features of the patients with advanced NSCLC

Characteristics	Number of cases (%)
Age	62 ± 12.04
< 65	10
≥ 65	8
Sex	
Male	14 (78)
Female	4 (22)
Stage	
IIIA	6 (33)
IIIB	6 (33)
IV	6 (33)
Pathological type	
Adenocarcinoma	8 (44)
Squamous cell carcinoma	9 (50)
Other	1 (6)
RECIST	
Complete response	1 (6)
Partial response	8 (44)
Stable disease	8 (44)
Progressive disease	1 (6)

### CCRT

Patients were treated with chemoradiotherapy in a concurrent regimen. An intensity-modulated radiotherapy technique (IMRT) or three-dimensional conformal RT (3D-CRT) was delivered to all patients with megavoltage equipment (6 MV). RT was given as the conventionally fractionated regimen, 1.8 to 2.0 Gy for five days per week, and the total dose administered to patients ranged from 56 to 66 Gy (median dose, 60 Gy). RT was planned based on a CT scan performed for planning purposes, the gross tumor volume (GTV) included the primary tumor and involved lymph nodes, and the planning target volume (PTV) included the GTV plus a margin of 1.0–1.5 cm. All patients were treated with two cycles of chemotherapy with a cisplatin/docetaxel or a cisplatin/pemetrexed region during RT and the first cycle of chemotherapy was applied on day 1 of RT. Two to four additional cycles of chemotherapy were needed every 3 weeks after RT.

### PET scanning

The simple lyophilized kit for labeling PRGD2 peptide was purchased from Jiangsu Institute of Nuclear Medicine, and the synthesis process was carried out by reference to previous study [13]. The radiochemical purity of the ^18^F-alfatide exceeded 95 %, and its specific radioactivity exceeded 37 GBq (1,000 mCi)/μmol. Patients were not requested to fast and to confirm blood glucose levels. After injected with^18^F-alfatide (214.38 ± 19.8 MBq) intravenously, they needed to rest for approximately 60 min. Scanning was performed with an integrated in-line PET/CT system (Discovery LS; GE Healthcare). PET images were performed from the head to the thigh, and the spiral CT component was performed with an x-ray tube voltage peak of 140 kV, 80 mA, a 6:1 pitch, a slice thickness of 4.25 mm, and a rotation speed of 0.8 s per rotation. A full-ring dedicated PET scan of the same axial range followed. The patients were in normal shallow respiration during image acquisition. The images were attenuation-corrected with the transmission data from CT. The attenuation-corrected PET images, CT images, and fused PET/CT images, displayed as coronal, sagittal, and transaxial slices, were viewed on a Xeleris workstation (GE Healthcare). The ^18^F-alfatide PET/CT scans were performed within 7 days before the start of CCRT.

### Image analysis

Two experienced nuclear medicine physicians assessed the ^18^F-alfatide PET/CT images visually, referring to PET fusion and CT images, until consensuses were reached. Acquired ^18^F-alfatide PET/CT data was transferred into the workstation in the DICOM format. The radiotracer concentration in the regions of interest (ROI) was normalized to the injected dose per kilogram of the patients’ body weight to derive the standardized uptake values (SUVs). The SUVs were calculated according to the following formula: [measured activity concentration (Bq/mL) × body weight (g)]/injected activity (Bq).

PET/CT parameters such as maximum and mean standard uptake values (SUV_max_ and SUV_mean_) and tumor volume (TV_PET_) were generated using a vendor-provided automated contouring program. Peak standard uptake values (SUV_peak_) were defined as the average SUV in a 1 cm^3^ sphere surrounding the voxel with the highest activity. We outlined the healthy lung with the position and volume similarly to the primary tumor to obtain the maximal activity of lung background. In addition, the maximal activity of 1 cm^3^ within the aortic arches and erector spinae were measured. Then the ratios of primary tumor and normal tissues based on SUV_max_ were calculated, denoted as T/NT (T/NT_lung_, T/NT_blood_ and T/NT_muscle_). In addition, tumor volumes were also measured by the CT images of PET/CT images, donated as TV_CT_.

### Response evaluation

Short-term outcome was assessed at 4 weeks after CCRT (56–66 Gy RT and 4–6 cycles of chemotherapy) according to the revised RECIST criteria (v.1.1) using chest CT. According to RECIST criteria, the responders included the patients with an outcome of complete response (CR) or partial response (PR); the patients who had an outcome of stable disease (SD) or progressive disease (PD) were classified as the non-responders.

### Statistical analysis

All statistical tests were performed with SPSS 17.0 and MedCalc 11.0.1.0. Statistical significance was assumed for P values less than 0.05 and all P values were 2-tailed. Eighteen patients were classified as responders and non-responders according to the revised RECIST criteria (v.1.1). Quantitative data for SUV_max_, SUV_peak_, SUV_mean_, TV_PET_, TV_CT_ and T/NT (T/NT_lung_, T/NT_blood_ and T/NT_muscle_) were expressed as mean ± standard deviation (SD) or median (interquartile range). Two-sample *t* tests and Wilcoxon rank-sum tests were used to compare the PET/CT parameters between responders and non-responders. SUV_max_, SUV_peak_ and T/NT and multiple clinical variables such as age, stage, and histopathology were tested by logistic regression analyses to identify the relationships between these variables and the short-term outcomes. Receiver-operating characteristic (ROC) curve analysis was used to achieve the thresholds with the maximum Youden index and determine the diagnostic accuracy of ^18^F-alfatide PET/CT parameters in identifying the responders and non-responders.

## Results

### Tumor response

Eighteen patients with advanced NSCLC had undergone ^18^F-alfatide PET/CT scans. Nine patients were classified as responders (52 %), including one complete response, eight partial responses, and nine patients were classified as non-responders (48 %) including eight stable disease and one progressive disease.

### Correlations between ^18^F-alfatide PET/CT parameters and tumor response

SUV_max_, SUV_peak_, SUV_mean_, TV (TV_PET_ and TV_CT_) and T/NT (T/NT_lung_, T/NT_blood_ and T/NT_muscle_) are listed in Table 2. The single data of SUV_max_, SUV_peak_ and SUV_mean_ are presented in Table 3. The differences of SUV_mean_, TV_PET_ and TV_CT_ between responders and non-responders were not significant in statistics (3.14 ± 0.17 vs. 3.76 ± 0.24, *P* = 0.05, 15,872 (27,232) vs. 55,296 (68,864), *P* = 0.07 and 32,856 (39,664) vs. 53,558 (90,508), P = 0.59). SUV_max_, SUV_peak_, T/NT_lung_, T/NT_blood_ and T/NT_muscle_ were significantly higher in non-responders than in responders (7.61 ± 0.77 vs. 4.95 ± 0.61, P = 0.024, 6.22 ± 0.65 vs. 3.99 ± 0.51, *P* = 0.016, 8.31 ± 0.61 vs. 6.53 ± 0.78, *P* < 0.001, 6.77 ± 0.63 vs. 3.86 ± 0.0.57, *P* = 0.003 and 12.56 ± 0.73 vs. 7.87 ± 1.14, *P* = 0.004).

**Table 2:** Parameters of pretreatment for^18^F-alfatide PET/CT scan

Parameters	All patients	Non-responders	Responders	p
SUV_max_	6.28 ± 2.44	7.61 ± 0.77	4.95 ± 0.61	0.024
SUV_mean_	3.44 ± 0.69	3.76 ± 0.24	3.14 ± 0.17	0.05
SUV_peak_	5.10 ± 2.06	6.22 ± 0.65	3.99 ± 0.51	0.016
TV_PET_	22,368 (62,480)	55,296 (68,864)	15,872 (27,232)	0.07
TV_CT_	37,570 (61,028)	53,558 (90,508)	32,856 (39,664)	0.59
T/NT_lung_	6.27 ± 2.5	8.31 ± 0.61	4.31 ± 0.48	<0.001
T/NT_blood_	5.51 ± 2.56	6.77 ± 0.63	3.86 ± 0.57	0.003
T/NT_muscle_	10.32 ± 3.53	12.56 ± 0.73	7.87 ± 1.14	0.04

**Table 3:** The single data of SUV_max_, SUV_peak_ and SUV_mean_

patients	SUV_max_	SUV_peak_	SUV_mean_
1	5.32	3.72	3.40
2	9.21	7.19	4.90
3	3.95	3.00	3.01
4	8.74	8.45	4.02
5	2.86	2.41	2.21
6	5.11	4.56	3.08
7	8.33	7.47	4.30
8	8.07	6.44	3.85
9	4.70	4.20	3.07
10	9.40	7.68	4.06
11	3.69	3.15	2.86
12	4.90	4.35	3.17
13	5.16	4.33	3.45
14	4.60	3.06	2.99
15	11.79	9.08	4.49
16	5.97	4.58	3.45
17	4.31	3.52	3.00
18	7.00	4.68	2.78

### ROC curve analysis

**Fig. 1:**
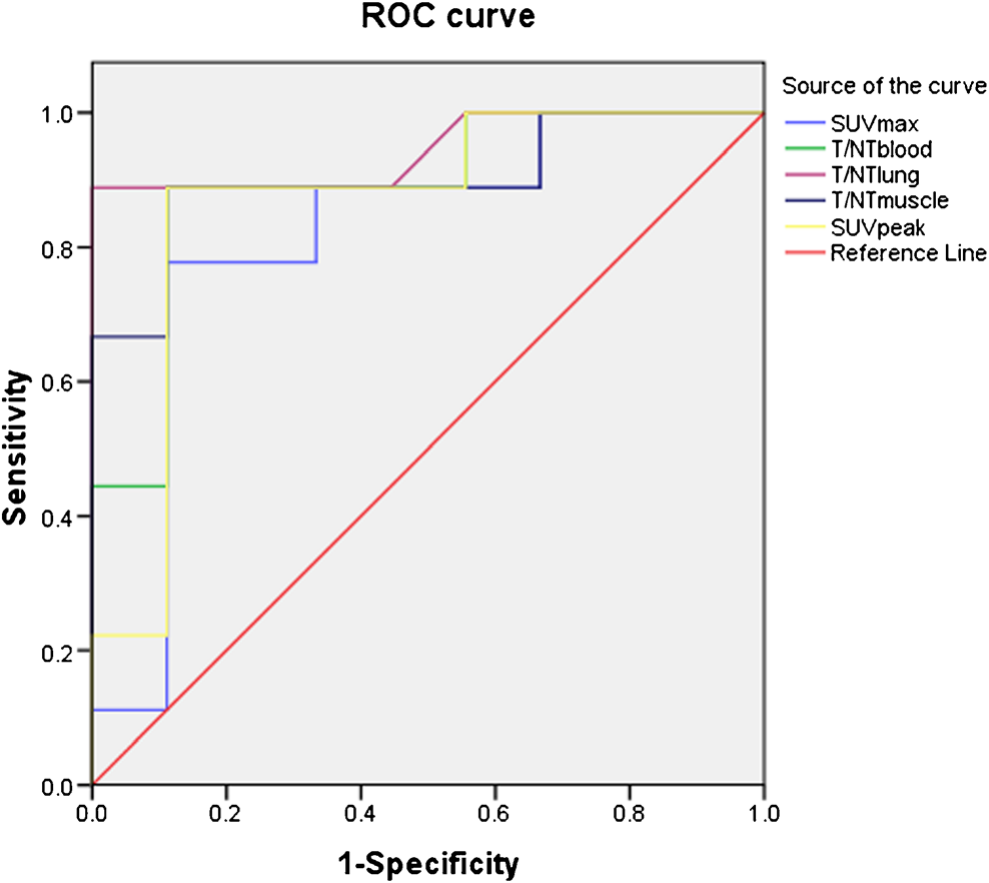
ROC curves of ^18^F-alfatide PET/CT parameters

ROC curve analysis was performed to determine the diagnostic accuracy of the five parameters (SUV_max_, SUV_peak_, T/NT_lung_, T/NT_blood_ and T/NT_muscle_) in identifying responders. There were highly significant correlations between SUV_max_, SUV_peak_, T/NT_lung_, T/NT_blood_ and T/NT_muscle_ of ^18^F-alfatide PET/CT and the short-term outcomes assessed by RECIST (*P* < 0.001) (Fig. 1). The AUC of T/NT_lung_ (AUC = 0.944) were higher than SUV_max_, SUV_peak_, T/NT_blood_ and T/NT_muscle_ (AUC = 0.815, 0.864, 0.889, 0.901) (Table 4), but the differences between them were not statistically significant (tested by MedCalc 11.0.1.0). According to ROC curve analysis, the thresholds of SUV_max_, SUV_peak_, T/NT_lung_, T/NT_blood_ and T/NT_muscle_ were 5.65, 4.46, 7.11, 5.41, and 11.75, respectively. The sensitivity, specificity, and accuracy of SUV_max_ for predicting tumor response were 77.8, 88.9, and 83.3 %, respectively. The sensitivity, specificity, and accuracy of T/NT_lung_ were 88.9, 100, and 94.4 %, respectively. The sensitivity, specificity, and accuracy of SUV_peak_,T/NT_blood_ and T/NT_muscle_ for predicting tumor response were all 88.9, 88.9, and 88.9 %, respectively (Table 5).

**Table 4:** Area under the curve of SUV_max_, SUV_peak_, T/NT_lung_, T/NT_blood_ and T/NT_muscle_ for predicting tumor response

Interval test result variable (s)	Area	SE^a^	Asymptotic sig.^b^	Asymptotic 95 % confidence interval	
				Lower bound	Upper bound
SUV_max_	0.815	0.901	0.079	0.517	1
SUV_peak_	0.864	0.096	0.009	0	1
T/NT_lung_	0.944	0.58	0.001	0	1
T/NT_blood_	0.889	0.081	0.005	0	1
T/NT_muscle_	0.901	0.079	0.004	0	1

^a^Under the nonparametric assumption

^b^Null hypothesis: true area = 0.5

**Table 5:** The specificity, sensitivity, and accuracy of SUV_max_, SUV_peak_, T/NT_lung_, T/NT_blood_ and T/NT_muscle_ for predicting tumor response

Parameters	Threshold	Sensitivity	Specificity	Accuracy
SUV_max_	5.65	77.8	88.9	88.9
SUV_peak_	4.46	88.9	88.9	88.9
T/NT_lung_	7.11	88.9	100	94.4
T/NT_blood_	5.41	88.9	88.9	88.9
T/NT_muscle_	11.75	88.9	88.9	88.9

### ^18^F-alfatide PET/CT parameters compared to other predictors

Multiple clinical variables included patients’ age, stage, histopathology, and ^18^F-alfatide PET/CT parameters (SUV_max_, SUV_peak_, T/NT_lung_, T/NT_blood_ and T/NT_muscle_) were tested by binary logistic regression analyses. We did not take smoking into account because only four patients had never smoked. According to univariate analyses, all the five ^18^F-alfatide PET/CT parameters that could predict the short-term outcome of CCRT, patients’ age, stage, and histopathology failed. Multivariate analyses were performed when baseline characteristics and ^18^F-alfatide PET/CT parameters (SUV_max_, SUV_peak_, T/NT_lung_, T/NT_blood_ and T/NT_muscle_, respectively) were considered. The result showed that T/NT_lung_ was a significant predictor of CCRT sensitivity (*P* = 0.032) based on binary logistic regression analyses. However, the SUV_max_ (*P* = 0.080), SUV_peak_ (*P* = 0.088), T/NT_blood_ (*P* = 0.098) and T/NT_muscle_ (*P* = 0.060) were not predictive for the short-term outcome of CCRT.

## Discussion

**Fig. 2:**
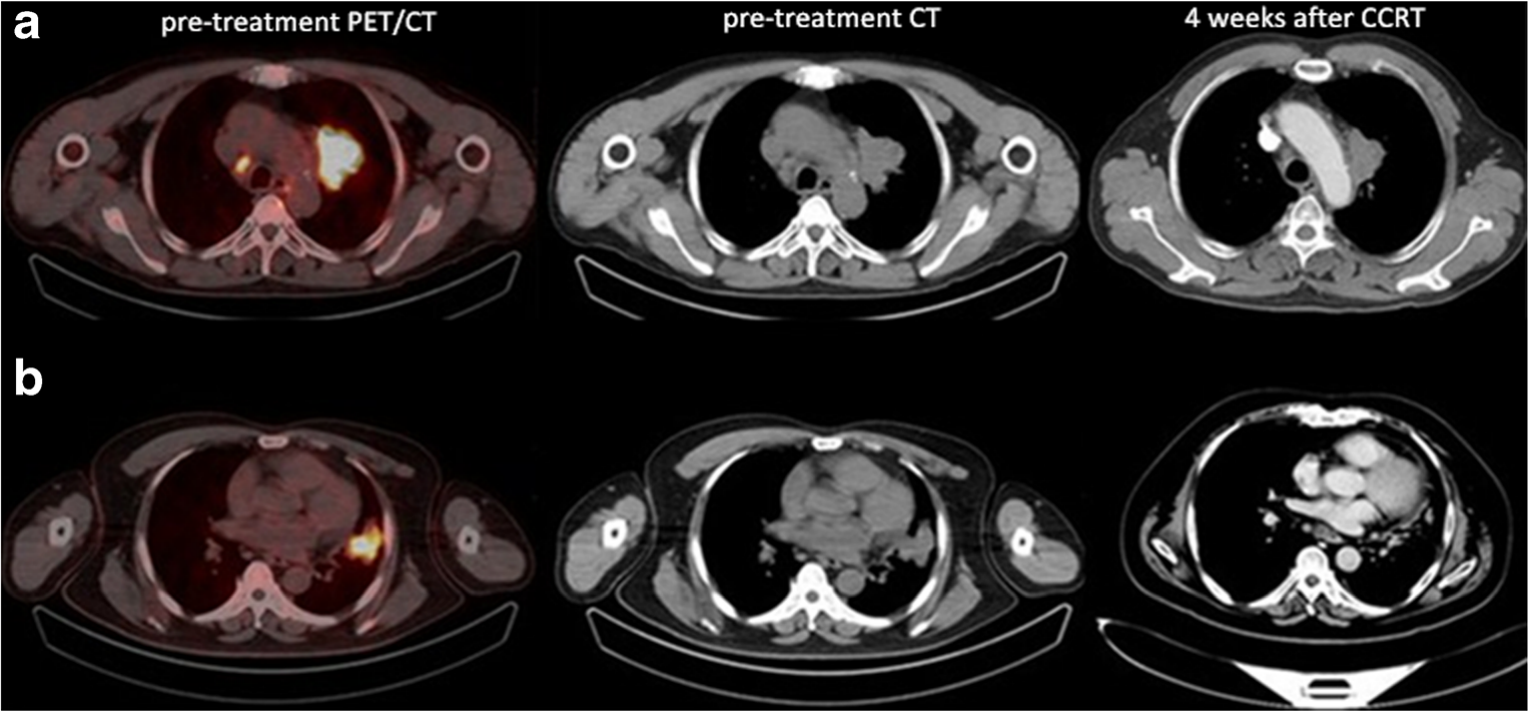
Two typical examples of ^18^F-alfatide PET/CT scans in patients with non-responding (**a**, T/NT_lung_ = 8.88) and responding (**b**, T/NT_lung_ = 6.47) tumors

There are significant differences in CCRT responses among the advanced NSCLC patients, so the early prognosis of the sensitivity to CCRT is the premise of personalized treatment. The TNM staging system and histopathology are considered important prognostic tools of overall survival, but they have limited effect on the short-term outcomes. In this study, the results indicated that ^18^F-alfatide PET/CT may be useful in predicting the short-term outcome of CCRT in patients with advanced NSCLC (Fig. 2). SUV_max_, SUV_peak_, T/NT_lung_, T/NT_blood_ and T/NT_muscle_ obtained from ^18^F-alfatide PET/CT were higher in non-responders than responders. Even in the multivariate logistic regression analyses, T/NT_lung_ was still an independent predictor of CCRT sensitivity.

PET is a non-invasive modality to evaluate specific molecular progress and a potential tool in the prediction of treatment response. Huang et al. found that the changes in SUV and metabolic tumor volume (MTV) obtained from ^18^F-FDG PET/CT of pre-treatment and intra-treatment CRT were significantly different between responders and non-responders in patients with locally advanced NSCLC (*P* = 0.002). However, the baseline parameters failed to differentiate the responders and the non-responders (all *P* > 0.05) [15]. A study showed that baseline ^18^F-fluorothymidine (FLT) PET achieved prediction of the treatment response in patients with lung cancer and non-Hodgkin lymphoma [16]. ^18^F-fluoromisonidazole (FMISO) PET, as an index of tissue oxygenation, can potentially aid in disease prognosis, given the leading role of hypoxia in radiation resistance [17]. Recently, our team had performed a pilot clinical study in which ^18^F-alfatide PET/CT parameters could predict the tumor sensitivity to CCRT in patients with glioma. Both baseline SUV_max_ and intra-treatment SUV_max_ showed correlations with response to CCRT, with the lesion volume change determined by MRI as the “gold” standard [14].

Why ^18^F-alfatide PET/CT is useful in prediction of the response to CCRT in patients with advanced NSCLC? ^18^F-alfatide PET/CT was known to be helpful to evaluate tumor angiogenesis, and angiogenesis was well recognized as an essential marker for tumor growth, invasion, and metastasis [18]. The integrin αvβ3 is up-regulated on the activated endothelial cells with tumor angiogenesis, and it can bind to ^18^F-alfatide with high affinity and specificity. Therefore, the ^18^F-alfatide uptake of tumor was potentially able to predict the responsiveness to CCRT in patients with advanced NSCLC. Similarly, Niu G and Chen X et al. commented that the responsiveness of glioma to CCRT may be partially due to the low malignancy indicated by the low SUV of ^18^F-alfatide PET/CT [19]. Besides, the differences between responders and non-responders to CCRT may be attributed to hypoxia. Neo-angiogenic vessels are often poorly perfused with low microvascular pressure, thus promoting blood stasis and hypoxia [20]. This can lead to suboptimal delivery of chemotherapy and also increases radio-resistance in tumors [21]. Therefore, the value of SUV obtained from ^18^F-alfatide PET/CT may be useful in predicting the short-term outcome of CCRT in patients with advanced NSCLC.

The results showed that SUV_mean_ and TV_PET_ were not different statistically significant between responders and non-responders. SUV_mean_ and TV_PET_ incorporate both tumor volume and metabolic activity, and they need accurate tumor contours. They could be easily affected by the setting threshold and the heterogenous uptake of the ^18^F-alfatide. That may be the reason why SUV_mean_ and TV_PET_ cannot respond to CCRT short-term outcome of the tumors as well as SUV_max_.

As ^18^F-alfatide PET/CT was helpful to evaluate the tumor angiogenesis, it may be potential in acting as a predictive biomarker to select patients who will most likely benefit from a specific angiogenesis inhibitor, and to detect emerging resistance. Hopefully, more clinical studies are needed to reveal the value of ^18^F-alfatide PET/CT in therapy decisions and for therapy response monitoring in these diseases.

## Conclusion

This study showed that ^18^F-alfatide PET/CT may be used to predict the short-term outcome of CCRT in patients with advanced NSCLC. With baseline SUV_max_, SUV_peak_ and T/NT, patients’ screening may be performed to avoid unnecessary therapy. The number of patients included in this study is small and a further validation study is needed to test the potential of ^18^F-alfatide PET/CT in guiding treatment decisions. In addition, it is a pity that only two patients received both ^18^F-alfatide PET/CT and ^18^F-FDG PET/CT scans, and we will continue to compare the potential ability of ^18^F-FDG PET/CT and ^18^F-alfatide PET/CT for prediction of the responses to CCRT in patients with advanced NSCLC.
